# Efficacy and workload analysis of a fixed vertical couch position technique and a fixed‐action–level protocol in whole‐breast radiotherapy

**DOI:** 10.1120/jacmp.v16i2.5265

**Published:** 2015-03-08

**Authors:** Saskia Petillion, Karolien Verhoeven, Caroline Weltens, Frank Van den Heuvel

**Affiliations:** ^1^ Department of Radiation‐Oncology University Hospitals of Leuven, KU Leuven Leuven Belgium; ^2^ Gray Institute for Radiation Oncology and Biology, University of Oxford Oxford UK

**Keywords:** breast radiotherapy, patient setup, off‐line correction, couch position, action level

## Abstract

Quantification of the setup errors is vital to define appropriate setup margins preventing geographical misses. The no‐action–level (NAL) correction protocol reduces the systematic setup errors and, hence, the setup margins. The manual entry of the setup corrections in the record‐and‐verify software, however, increases the susceptibility of the NAL protocol to human errors. Moreover, the impact of the skin mobility on the anteroposterior patient setup reproducibility in whole‐breast radiotherapy (WBRT) is unknown. In this study, we therefore investigated the potential of fixed vertical couch position‐based patient setup in WBRT. The possibility to introduce a threshold for correction of the systematic setup errors was also explored. We measured the anteroposterior, mediolateral, and superior–inferior setup errors during fractions 1–12 and weekly thereafter with tangential angled single modality paired imaging. These setup data were used to simulate the residual setup errors of the NAL protocol, the fixed vertical couch position protocol, and the fixed‐action–level protocol with different correction thresholds. Population statistics of the setup errors of 20 breast cancer patients and 20 breast cancer patients with additional regional lymph node (LN) irradiation were calculated to determine the setup margins of each off‐line correction protocol. Our data showed the potential of the fixed vertical couch position protocol to restrict the systematic and random anteroposterior residual setup errors to 1.8 mm and 2.2 mm, respectively. Compared to the NAL protocol, a correction threshold of 2.5 mm reduced the frequency of mediolateral and superior–inferior setup corrections with 40% and 63%, respectively. The implementation of the correction threshold did not deteriorate the accuracy of the off‐line setup correction compared to the NAL protocol. The combination of the fixed vertical couch position protocol, for correction of the anteroposterior setup error, and the fixed‐action–level protocol with 2.5 mm correction threshold, for correction of the mediolateral and the superior–inferior setup errors, was proved to provide adequate and comparable patient setup accuracy in WBRT and WBRT with additional LN irradiation.

PACS numbers: 87.53.Kn, 87.57.‐s

## I. INTRODUCTION

The standard treatment of early‐stage breast cancer is breast‐conserving surgery followed by fractionated conformal whole‐breast radiotherapy (WBRT). Regional lymph node (LN) irradiation in addition to WBRT (i.e., WBRT‐LN) improves disease‐free and metastases‐free survival of patients with stage I‐III breast cancer.^(1)^ Variations in daily patient setup are compensated by prescribing the dose to the planning target volume (PTV) (i.e., a 3D expansion of the clinical target volume (CTV)).^(2)^ Large PTV margins, however, increase the dose delivered to the surrounding healthy tissues. PTV margins can be limited predominantly by reducing the systematic setup error.^(3)^ Several off‐line correction protocols have been proposed to correct the systematic setup errors using quantitative analysis of images. In the early days of portal imaging, Bel et al.^(4)^ introduced the shrinking‐action–level (SAL) protocol. Large systematic setup errors are corrected at an early stage of the treatment by decreasing the correction threshold with increasing number of measurements.^(4)^ However, its high resulting setup accuracy is obtained after a variable and possibly high number of imaged fractions and requires a variable number of setup corrections, increasing both the healthy tissue dose and the workload. de Boer and Heijmen,^(5)^ therefore, proposed the no‐action–level (NAL) protocol. Each systematic setup error is estimated and corrected after a fixed number of imaged fractions. With Monte Carlo simulations they have shown that to halve the systematic setup error, the NAL protocol only requires 3–5 imaged fractions, while the SAL protocol needs 8–9 imaged fractions. Due to this superiority, the NAL protocol has an extensive application in WBRT with high resulting setup accuracy.^6^, ^7^, ^8^

In single‐isocentric WBRT and WBRT‐LN, patient setup using four skin marks is followed by a 3D couch shift to position the breast in the treatment isocenter. With the current advances in treatment couch technology and record‐and‐verify software, the planned couch shifts are performed automatically. In contrast, setup corrections determined by the NAL protocol have to be entered manually. Hence, considering the large breast cancer population, it is vital to restrict the workload and the susceptibility of the off‐line setup correction protocol to human errors by reducing the number of corrections without losing on the accuracy. The implementation of a threshold for setup correction in breast radiotherapy has, however, never been investigated. Moreover, the impact of the skin mobility on the anteroposterior patient setup reproducibility in breast radiotherapy is unexplored. From pelvic radiotherapy, it is however known that fixed vertical couch setup restricts the impact.^(9)^

In this paper, we therefore first investigated the impact of the skin mobility on the anteroposterior setup error in WBRT by comparing the accuracy of the fixed vertical couch position protocol to the accuracy of the NAL protocol. Secondly, we explored the potential of a fixed‐action–level (FAL) protocol to reduce the workload while maintaining setup accuracy in WBRT. Different thresholds for setup correction were investigated. The clinically obtained accuracy of one of the investigated off‐line correction protocols was verified in WBRT and WBRT‐LN.

## II. MATERIALS AND METHODS

### A. Whole‐breast radiotherapy with and without additional lymph node irradiation

Twenty consecutive WBRT patients (median age: 61 yrs, range: 43–88 yrs) and 20 consecutive WBRT‐LN patients (median age: 55 yrs, range: 42–77 yrs) were scanned in the treatment position: supine on a Posiboard‐2 breastboard (Civco Medical Solutions, Orange City, IA), with the arms raised above the head and with an immobilization wedge under the knees. A free‐breathing CT scan was acquired (Somatom Sensation Open, Siemens Medical Solutions, Erlangen, Germany). Four skin marks defined the patient setup position. Three were in an axial plane at half the height of the clinical breast volume: one on the sternum and one on each lateral side of the patient. The fourth skin mark was about 15 cm caudal to the sternum on the sagittal laser line.

WBRT was performed with single‐isocentric, two‐field, forward‐planned IMRT to a total dose of 42.56 Gy or 50 Gy in 16 or 25 daily fractions, respectively. The single‐isocentric, four‐field oblique parasternal photon^(10)^ or three‐field partly wide tangential technique^(11)^ was used for WBRT‐LN to a total dose of 50 Gy in 25 daily fractions. The breast tissue (${\text{CTV}}_{\text{Breast}}$) and, in case of WBRT‐LN, the internal mammary‐medial supraclavicular LNs (${\text{CTV}}_{\text{IMMS}}$) were delineated by the same experienced physician. The PTV margins prescribed in the EORTC 22922‐10925 trial^(1)^ were applied (i.e., 10 mm around ${\text{CTV}}_{\text{Breast}}$ and 5 mm around ${\text{CTV}}_{\text{IMMS}}$). The isocenter and field setup were determined during virtual simulation.

All treatments were delivered on linear accelerators equipped with on‐board imaging (OBI) (Varian Medical Systems, Inc., Palo Alto, CA). Daily patient setup consisted of aligning the skin marks with the treatment room lasers followed by a 3D couch shift to position the breast in the treatment isocenter.

### B. Online setup verification and correction

**Figure 1:**
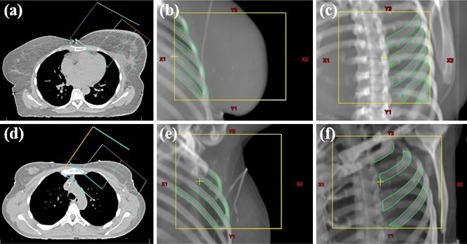
Tangential angled kV‐kV imaging in left‐sided WBRT (upper row) and left‐sided WBRT‐LN (lower row): setup ((a),(d)) of the localization fields in the direction of the medial tangential beam ((b),(e)) and orthogonal to the irradiation direction ((c),(f)).

The setup error (i.e., the deviation between the intended and the actual patient setup) was determined using tangential angled kV‐kV imaging.^(12)^ Two orthogonal kV images were acquired and matched to the corresponding digitally reconstructed radiograph using the ribs. In case of left‐sided breast irradiation, the first kV image was acquired in the direction of the medial tangential beam (Fig. 1). For right‐sided breast cancer patients, the first kV image was acquired in the direction of the lateral tangential beam. This technique was proven to be robust to breathing motion.^(12)^ Moreover, tangential angled kV‐kV–based setup correction has comparable accuracy in WBRT and WBRT‐LN.^(12)^ The anteroposterior (AP), mediolateral (ML), and superior–inferior (SI) setup errors were measured and corrected during fractions 1–12 and weekly thereafter. Moreover, the vertical couch position after initial patient setup was recorded daily.

### C. Simulated residual setup errors of different off‐line setup correction protocols applied in WBRT

The measured setup errors of fractions 1–5 in WBRT and WBRT‐LN were used to calculate the patient's average AP AP(±standard deviation(SD)),ML(±SD), and SI(±SD) setup error. de Boer and Heijmen^(5)^ reported 5 to be the optimal number of imaged fractions to estimate the patient's systematic (i.e., average, setup error). Moreover, the vertical couch treatment position was calculated as:

(1) $${\text{Couch}}_{\text{Vrt},\text{RT},i}={\text{Couch}}_{\text{Vrt},i}+{\text{SE}}_{\text{AP},i}$$

with fraction *i* ∈ [1,5]; $i\in [1,5];Couch_{Vrt,i}=$ the vertical couch position after initial patient setup during fraction *i*; and $SE_{AP,i}=$ the measured *AP* setup error of fraction *i*.

The average setup errors were used for patient setup from fraction 6 to correct the systematic AP, ML, and SI setup errors if ≥2.5 mm. These corrections were added to the measured residual setup errors of fractions 6–10 to obtain the total setup errors. These uncorrected setup data were then used to simulate the residual setup errors of 1) the no‐action–level (NAL) protocol, 2) the fixed vertical couch position (FVCP) protocol, and 3) the fixed‐action‐level (FAL) protocol with different setup correction thresholds. The simulated residual setup error is the deviation between the intended patient setup and the simulated patient setup using one of the off‐line correction protocols.

#### C.1 No‐action–level protocol

The NAL protocol corrects each patient's systematic setup error without threshold. For fractions 6–10, we simulated the AP, ML, and SI residual setup errors that would have occurred if the NAL protocol would have been applied by

(2) $${\text{RE}}_{\text{NAL},x,i}={\text{SE}}_{x,i}-\sum_{x,1-5}$$

with x=AP, ML, or SI; fraction $i\in [6,10];SE_{x,i}=$ the total setup error in direction *x* of fraction i; and $\Sigma_{x,1-5}=$ the patient's systematic setup error of fractions 1‐5 in direction x.

#### C.2 Fixed vertical couch position protocol

We explored the impact of applying a fixed vertical couch position by first calculating the average vertical couch treatment position of fractions 1‐5 (i.e., ${\text{Couch}}_{\text{Vrt},\text{RT},\text{Avg}}$). Next, for fractions 6–10, we simulated the AP residual setup error that would have occurred if ${\text{Couch}}_{\text{Vrt},\text{RT},\text{Avg}}$ would have been applied. Therefore, ${\text{Couch}}_{\text{Vrt},\text{RT},\text{Avg}}$ was subtracted from the vertical couch treatment position of fractions 6–10. The latter is the vertical couch position after correcting the total AP setup error of fractions 6–10. Hence, the residual setup error of the fixed vertical couch position protocol was simulated by

(3) $${\text{RE}}_{\text{FVCP},i}=({\text{Couch}}_{\text{Vrt},i}+{\text{SE}}_{\text{AP},i})-{\text{Couch}}_{\text{Vrt},\text{RT},\text{Avg}}$$

with fraction $i\in [6,10];Couch_{Vrt,i}=$ the vertical couch position after initial patient setup during fraction *i* (i.e., with the clinically applied patient's systematic setup correction eliminated (see above)); $SE_{AP,i}=$ the total AP setup error of fraction *i*; and $Couch_{Vrt,RT,Avg}=$ the average vertical couch treatment position of fractions 1–5.

#### C.3 Fixed‐action–level protocol

As an alternative for the NAL protocol, we investigated the FAL protocol with different correction thresholds (i.e., ${\text{FAL}}_{y}$) with y=1.5 mm,2.5 mm,3.5 mm, and 4.5 mm. Starting at fraction 6, the patient's systematic setup errors were corrected if larger than the correction threshold. We simulated the residual setup errors that would have occurred if the ${\text{FAL}}_{y}$ protocol was applied by

(4) $${\text{RE}}_{\text{FALy},x,i}={\text{SE}}_{x,i}-\sum_{x,1-5}$$

with x=AP, ML, or SI; fraction $i\in [6,10];\Sigma_{x,1-5}=$ the patient's systematic setup error of fractions 1–5 ≥ threshold *y* ($RE_{FALy,x,i}=SE_{x,i}$ if $\Sigma_{1-5}<\text{threshold}$ *y).*

### D. Comparison of the accuracy of the different off‐line setup correction protocols applied in WBRT

The patient's average AP, ML, and SI setup errors (±1 SD) of fractions 1–5 yielded the patient's systematic (± random) setup errors if no off‐line setup correction protocol would be applied. The patient's average AP, ML, and SI setup errors (±1 SD) of fractions 6–10 yielded the patient's systematic (± random) residual setup errors of the off‐line correction protocols under investigation.

To assess the accuracy of each off‐line setup correction protocol, we calculated the population mean $\left(\mu_{P}\right)$, the systematic $\left(\Sigma_{P}\right)$, and the random $\left(\sigma_{P}\right)$ setup errors of fractions 1–5 (no off‐line setup correction) and fractions 6–10 (NAL protocol, FVCP protocol, and ${\text{FAL}}_{y}$ protocol). The population mean setup error $\left(\mu_{P}\right)$ was calculated as the average of the patient systematic setup errors.^(13)^ The systematic setup error $\left(\Sigma_{P}\right)$ was calculated as the SD of the patient average setup errors and the random setup error $\left(\sigma_{P}\right)$ as the root mean square of the patient random setup errors.^(13)^ In this study, we focused on the reduction of the systematic setup errors and checked the differences between the off‐line setup correction protocols for significance with the chi‐squared test. Differences were considered significant if p<0.05. As the chi‐squared test was performed twice on the AP component of the setup error, we performed a Bonferroni correction by adjusting the significance level to 0.05/2.^(14)^ Hence, differences in the AP component were considered significant if p<0.05/2.

Based on the measured setup errors (fractions 1–5) and the simulated residual setup errors (fractions 6–10), we calculated the PTV margins needed to account for the (residual) setup errors before and after application of the off‐line setup correction protocols under investigation. We used a margin recipe of van Herk et al.:^(3)^ $\text{PTV}=2.5\;\Sigma_{P}+0.7\;\sigma_{P}$, with Σ_P_ the systematic and $\sigma_{P}$ the random setup error.

We also determined the number of corrections per threshold y of the ${\text{FAL}}_{y}$ protocol. The most optimal threshold was identified by minimizing the absolute value of the difference between the PTV margin of the NAL protocol and the PTV margin of the ${\text{FAL}}_{y}$ protocol (i.e., $\min(|{\text{PTV}}_{\text{NAL}}-{\text{PTV}}_{\text{FALy}}|)$). The PTVs of all components of the residual setup error were used in the minimization process to select one threshold that is applicable for each direction.

### E. Measured residual setup errors of one off‐line setup correction protocol applied in WBRT and WBRT‐LN

In clinical routine, the $\text{FVCP}-{\text{FAL}}_{2.5\text{mm}}$ protocol was applied in WBRT, starting at fraction 11, and in WBRT‐LN, starting at fraction 6. Hence, the AP patient setup was performed by raising the treatment couch to the fixed vertical couch position $\left({\text{Couch}}_{\text{Vrt},\text{RT},\text{Avg}}\right)$. The systematic ML and SI setup errors were corrected if ≥2.5 mm. The residual setup errors were measured and corrected during additional online setup verifications (see Materials & Methods section B).

In WBRT, online setup verification and correction was continued until all components of the residual setup error were between ±5 mm on two consecutive days. Otherwise, if the residual setup errors had identical sign on two consecutive days, a new correction of the patient's systematic setup error was calculated, applied, and online verified. Afterwards, online setup verification and correction was performed weekly, yielding on average 4 (range 3–8) additional imaged fractions per patient.

In WBRT‐LN, online setup verification and correction was continued till fraction 12 and weekly thereafter. For this study, only the measured residual setup errors of fractions 6–10 were used.

For WBRT and WBRT‐LN, the measured systematic $\left(\Sigma_{P}\right)$ and random $\left(\sigma_{P}\right)$ residual setup errors with associated PTV margin were calculated, as described in Materials & Methods section D, and compared to the simulated values of the $\text{FVCP}-{\text{FAL}}_{2.5\text{mm}}$ protocol. Moreover, we also determined the number of clinically applied off‐line setup corrections in WBRT‐LN.

## III. RESULTS

### A. Simulated residual setup errors of the fixed vertical couch position protocol applied in WBRT

**Figure 2:**
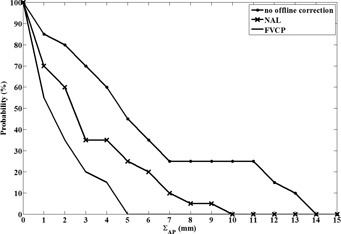
Cumulative frequency distribution of the patient's measured (no off‐line correction) and simulated (NAL and FVCP) systematic anteroposterior $\left(\Sigma_{\text{AP}}\right)$ (residual) setup error (N=20).

Figure 2 shows that before setup correction the patient's systematic AP setup error was ≥5 mm with a probability of 45%. Applying the NAL protocol reduced this probability to 25%. The maximum systematic AP residual setup error was 4 mm if the FVCP protocol was applied.

Table 1 reveals that omitting off‐line setup correction results in a large systematic AP setup error (5.7 mm) which was decreased to a residual setup error of 4.1 mm with the NAL protocol.

The 1.6 mm decrease was borderline significant (p=0.0158). The FVCP protocol was effective in significantly reducing the systematic AP residual setup error of the NAL protocol from 4.1 mm to 1.8 mm $\left(p<10^{-4}\right)$.

**Table 1:** Measured anteroposterior setup errors without off‐line setup correction during fractions 1–5 in WBRT. Simulated anteroposterior residual setup errors and number of corrections obtained for two offline correction protocols applied during fractions 6–10 in WBRT

	*No Off‐line Correction* (N=20)	*NAL* (N=20)	*FVCP* (N=20)
$\mu_{P}$ (mm)	−4.3	−4.3	0.5
$\Sigma_{P}$ (mm)	5.7	4.1	1.8
$\sigma_{P}$ (mm)	4.6	3.4	2.2
PTV (mm)	17.5	12.7	6.0
Number of corrections	0	20	20

WBRT= whole‐breast radiotherapy; $\mu_{P}=$ population mean setup error; $\Sigma_{P}=$ systematic setup error; $\sigma_{P}=$ random setup error; PTV= planning target volume; NAL= no‐action‐level; FVCP= fixed vertical couch position.

The NAL protocol reduced the random AP setup error from 4.6 mm to 3.4 mm. As expected, this reduction was not significant (p=0.0692, paired *t*‐test using a significance level of p<0.05/2 to include the Bonferroni correction for multiple testing^(14)^). In contrast, the FVCP protocol significantly reduced the random AP setup error from 4.6 mm to 2.2 mm ($p>10^{-3}$, paired *t*‐test using a significance level of p<0.05/2 to include the Bonferroni correction for multiple testing^(14)^) (Table 1). As a result, the FVCP protocol reduced the AP PTV margin with 11.5 mm (Table 1). In contrast, the NAL protocol reduced the PTV margin with only 4.8 mm (Table 1).

As the FVCP protocol is shown to be more effective in reducing the systematic and the random AP setup error than the NAL protocol, the NAL protocol was not retained for the correction of the AP setup error.

### B. Simulated residual setup errors of the fixed‐action–level protocol applied in WBRT

We did not explore the potential of a correction threshold for the FVCP protocol. Hence, to accurately correct the systematic AP setup error starting from fraction 6, we propose the FVCP protocol to be applied for all patients. This means that for each patient, the planned isocenter shift has to be deleted in the record‐and‐verify software and the treatment couch has to be raised to ${\text{Couch}}_{\text{Vrt},\text{RT},\text{Avg}}$.

**Figure 3:**
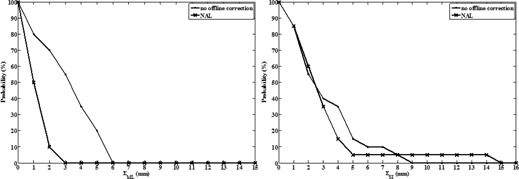
Cumulative frequency distributions of the patient's measured (no off‐line correction) and simulated (NAL) systematic mediolateral and superior–inferior (residual) setup errors ($\Sigma_{\text{ML}}$ and $\Sigma_{\text{SI}}$, respectively) (N=20).

Figure 3 shows the cumulative frequency distribution of the patient's systematic ML and SI residual setup errors of the NAL protocol. One patient had a systematic SI residual error >14 mm. The patient was excluded for further analysis as this systematic residual setup error was not representative for the expected accuracy of the NAL protocol. As mentioned in Materials & Methods section E, irrespective of the applied off‐line correction protocol, online setup verification and correction is continued until all components of the residual setup error are between ±5 mm on two consecutive days. Hence, the expected patient's systematic residual setup error of the NAL protocol is ≤5 mm. This is confirmed by the results of the other patients (Fig. 3).

As expected, the NAL protocol did not alter the random ML and SI setup errors, but significantly reduced the systematic ML setup error from 3.4 mm (no off‐line correction) to 1.3 mm $\left(p<10^{-4}\right)$, halving the ML PTV margin (Table 2). In contrast, the systematic SI setup error was not significantly reduced (p=0.3376) (Table 2). The PTV margins needed to account for the ML and SI residual setup errors were 5 mm and 9 mm, respectively.

**Table 2:** Measured mediolateral and superior–inferior setup errors without off‐line setup correction during fractions 1–5 in WBRT. Simulated mediolateral and superior–inferior residual setup errors and number of corrections obtained for different off‐line setup correction protocols applied during fractions 6–10 in WBRT

	*ML* (N=20)						*SI* (N=19)					
	*No Off‐line Correction*	*NAL*		*FAL*			*No Off‐line Correction*	*NAL*		*FAL*		
Threshold (mm)	–	0.0	1.5	2.5	3.5	4.5	–	0.0	1.5	2.5	3.5	4.5
$\mu_{P}$ (mm)	−0.02	0.2	0.02	−0.2	0.1	0.1	1.0	−0.08	−0.3	0.1	0.1	0.6
$\Sigma_{P}$ (mm)	3.4	1.3	1.4	1.3	1.6	2.2	3.3	2.7	2.7	2.6	2.6	3.1
$\sigma_{P}$ (mm)	2.3	2.6	2.6	2.6	2.6	2.6	3.1	3.3	3.3	3.3	3.3	3.3
PTV (mm)	10.1	5.1	5.4	5.1	5.7	7.4	10.4	9.2	9.0	8.8	8.8	10.2
Number of Corrections	0	20	14	12	8	4	0	19	12	7	7	3

WBRT= whole‐breast radiotherapy; $\mu_{P}=$ population mean setup error; $\Sigma_{P}=$ systematic setup error; σ_P_ = random setup error; PTV = planning target volume; ML = mediolateral; SI = superior‐inferior; NAL = no‐action‐level; FAL = fixed action level.

The most optimal threshold for the ${\text{FAL}}_{y}$ protocol is 2.5 mm as this does not alter the ML PTV margin (Table 2). The impact on the SI PTV margin is negligible, −0.4 mm (Table 2). The application of the ${\text{FAL}}_{2.5\text{mm}}$ protocol halved the number of manual ML and SI adjustments in the record‐and‐verify software compared to the NAL protocol (i.e., 19/39 (48.7%) corrections were required). Six patients (31.6%) did not require a correction of the systematic ML and SI setup errors. Five patients (26.3%) required the correction of both systematic setup errors.

In total, the application of the $\text{FVCP}-{\text{FAL}}_{2.5\text{mm}}$ protocol required 39/59 (66.1%) manual adjustments in the record‐and‐verify software, which is a reduction of 20/59 (33.9%) compared to the NAL protocol.

### C. Measured residual setup errors of the $\text{FVCP}-{\text{FAL}}_{2.5\text{mm}}$ protocol

#### C.1 In WBRT

The measured systematic AP, ML, and SI residual setup errors of the $\text{FVCP}-{\text{FAL}}_{2.5\text{mm}}$ protocol (2.0 mm, 1.7 mm, and 2.9 mm, respectively) were comparable to the simulated systematic residual setup errors (1.8 mm, 1.3 mm, 2.6 mm, respectively) (1, 2). Moreover, the measured (2.4 mm, 2.3 mm, 3.2 mm) and simulated (2.2 mm, 2.6 mm, 3.3 mm) random AP, ML, and SI residual setup errors, respectively, were found to be equivalent. This yielded the simulated AP (6 mm), ML (5 mm), and SI (9 mm) PTV margins (1, 2) to be comparable to the clinically required AP (7 mm), ML (6 mm), and SI (9 mm) PTV margins for the $\text{FVCP}-{\text{FAL}}_{2.5\text{mm}}$ protocol.

#### C.2 In WBRT‐LN

The measured systematic AP, ML, and SI residual setup errors of the $\text{FVCP}-{\text{FAL}}_{2.5\text{mm}}$ protocol in WBRT‐LN (2.0 mm, 1.3 mm, 2.7 mm, respectively (Table 3)) compared well to the simulated systematic residual setup errors in WBRT (1, 2). Moreover, the random AP, ML, and SI residual setup errors (3.0 mm, 2.8 mm, 3.1 mm (Table 3)) were equivalent to the simulations (1, 2). The anisotropic PTV margin associated with the measured residual setup errors (AP: 7 mm, ML: 5 mm, and SI: 9 mm) concurred with the simulated anisotropic PTV margin in WBRT (1, 2).

The $\text{FVCP}-{\text{FAL}}_{2.5\text{mm}}$ protocol required 20 manual adjustments of the AP isocenter shift, 11 of the ML isocenter shift and 7 of the SI isocenter shift (i.e., 38/60 (63.3%) manual adjustments). This corresponded well to the expected correction frequency (66.1%). Comparable to WBRT, 6/20 (30%) patients did not require a correction of the systematic ML and the SI setup errors. Moreover, 5/20 (20%) patients required the correction of both systematic setup errors.

**Table 3:** Measured setup errors (fractions 1–5), residual setup errors (fractions 6–10), and number of corrections of the $\text{FVCP}-{\text{FAL}}_{2.5\text{mm}}$ protocol applied in WBRT‐LN (N=20)

	*AP*		*ML*		*SI*	
	*No Offline Correction*	*FVCP*	*No Offline Correction*	*FAL*	*No Offline Correction*	*FAL*
Threshold Σ (mm)	–	0.0	–	2.5	–	2.5
$\mu_{P}$ (mm)	−5.8	0.5	−0.6	−0.4	1.8	−0.3
$\Sigma_{P}$ (mm)	6.0	2.0	2.9	1.3	2.5	2.7
$\sigma_{P}$ (mm)	5.1	3.0	2.7	2.8	3.0	3.1
PTV (mm)	19	7	9	5	8	9
Number of corrections	0	20	0	11	0	7

$\text{FVCP}-{\text{FAL}}_{2.5\text{mm}}=$ fixed vertical couch position – fixed‐action–level protocol with 2.5 mm correction threshold; WBRT−LN= whole‐breast radiotherapy with additional lymph node irradiation; AP= anteroposterior; ML= mediolateral; SI= superior‐inferior; $\mu_{P}=$ population mean setup error; $\Sigma_{P}=$ systematic setup error; $\sigma_{P}=$ random setup error; PTV= planning target volume.

## IV. DISCUSSION

Our data demonstrate the potential of a new off‐line setup correction protocol to restrict the PTV margins and reduce the workload in WBRT and WBRT‐LN. The mediolateral and superior‐inferior online setup measurements and the vertical couch treatment positions of fractions 1‐5 are used to calculate the mediolateral and superior‐inferior systematic setup errors and the average vertical couch treatment position $\left({\text{Couch}}_{\text{Vrt},\text{RT},\text{Avg}}\right)$, respectively. These data are then used for patient setup from fraction 6. Application of the fixed vertical couch position (FVCP) technique (i.e., raising the couch to ${\text{Couch}}_{\text{Vrt},\text{RT},\text{Avg}}$) restricts the anteroposterior PTV margin by significantly, reducing both the systematic and the random anteroposterior setup errors. Correction of the mediolateral and superior‐inferior setup errors using the fixed‐action‐level protocol with 2.5 mm correction threshold $\left({\text{FAL}}_{2.5\text{mm}}\right)$ achieves accuracy comparable to the NAL protocol. The proposed $\text{FVCP}-{\text{FAL}}_{2.5\text{mm}}$ protocol significantly reduces the frequency of manual adjustments in the record‐and‐verify software with at least 34% compared to the NAL protocol.

In agreement to the literature,^6^, ^7^, ^8^ we found the largest systematic setup error in its anteroposterior component. Our systematic anteroposterior residual setup error of the NAL protocol (4.1 mm) was larger than reported in previous studies:^6^, ^7^, ^8^ 1.7 mm, 2.1 mm, and 1.7 mm, respectively. However, with the FVCP protocol, we did obtain a systematic anteroposterior residual setup error (1.8 mm), comparable to the literature. Moreover, in contrast to the NAL protocol, the FVCP protocol also significantly reduced the random anteroposterior residual setup error. These findings have to be attributed to the mobility of the lateral skin marks with respect to the ribs, used as a surrogate of the breast position during online setup verification. In analogy with Veldeman et al.,^(15)^ the population means of the anteroposterior setup errors showed a negative offset between the actual and the intended isocenter position (1, 3). This reflects that the actual isocenter position was too much anterior relative to the intended isocenter position. This is caused by the patient's relaxation as she feels more comfortable during the course of treatment.^15^, ^16^ The skin mobility makes the lateral skin marks less reliable for anteroposterior patient setup. We showed that the skin mark movement is countered by the application of a fixed vertical couch position. This is in agreement with Roels et al.^(9)^ who proposed its implementation in rectal radiotherapy. Hence, our results reveal that, also in breast radiotherapy, there is evidence for a relative constant position of the bony anatomy with respect to the couch.

We propose the FVCP protocol to be applied for each breast cancer patient without correction threshold. For the mediolateral and the superior–inferior component of the setup error, we explored the potential of the implementation of a correction threshold to reduce the workload without deteriorating the setup accuracy. The ${\text{FAL}}_{2.5\text{mm}}$ protocol reduced the frequency of manual adjustments of the mediolateral and superior–inferior couch shifts in the record‐and‐verify software, compared to the NAL protocol with 40% and 63%, respectively. This restricts the susceptibility of the off‐line setup correction method to human errors. To our knowledge, we were the first to explore the potential of reducing the number of off‐line setup corrections in WBRT and WBRT‐LN. However, by application of the proposed $\text{FVCP}-{\text{FAL}}_{2.5\text{mm}}$ protocol to an independent patient group, we were able to confirm the correction reductions in mediolateral and superior–inferior direction, 45% and 65%, respectively. The proposed 2.5 mm correction threshold corresponds to the 3 mm correction threshold used by Roels et al.^(9)^ It excludes the correction of systematic setup errors which are related to clinically acceptable mechanical inaccuracies (couch movement accuracy, correspondence between OBI isocenter and treatment isocenter, image quality).

Our mediolateral and superior–inferior components of the residual setup error show that the ${\text{FAL}}_{2.5\text{mm}}$ protocol has comparable accuracy to the NAL protocol. This is in agreement to the simulation performed by de Boer and Heijmen^(5)^ for a 1 mm correction threshold. The systematic mediolateral residual setup error of the ${\text{FAL}}_{2.5\text{mm}}$ protocol (1.3 mm) compares well to the accuracy of the NAL protocol reported in literature: 1.7 mm,^(6)^ 1.7 mm,^(7)^ and 1.7 mm.^(8)^ Our systematic superior‐inferior residual setup error (2.6 mm) compares well to the systematic superior–inferior residual setup error reported by Harris et al.^(8)^ (2.0 mm), but is larger than reported by Penninkhof et al.^(6)^ (1.4 mm) and Lozano et al.^(7)^ (0.9 mm). These interstudy deviations are caused by the differences in the used immobilization devices and the applied patient setup protocol.

The simulated residual setup errors of the $\text{FVCP}-{\text{FAL}}_{2.5\text{mm}}$ protocol were confirmed by our clinical measurements in WBRT and WBRT‐LN. We therefore propose 7 mm, 5 mm, and 9 mm PTV margins in the anteroposterior, mediolateral, and superior–inferior direction, respectively, to be used in WBRT and WBRT‐LN, provided the proposed $\text{FVCP}-{\text{FAL}}_{2.5\text{mm}}$ off‐line setup correction protocol is applied. Compared to the PTV margins prescribed by the EORTC 22922‐10925 trial^(1)^ (1 cm for the breast and 0.5 cm for the LNs), we thus obtained a reduction of the in‐plane breast PTV margin. As in the study by Nielsen et al.,^(17)^ we found the largest systematic residual setup error in the superior–inferior direction. However, the 9 mm superior–inferior PTV margin is acceptable in WBRT, since this will not add dose to the organs at risk, being the heart and the lungs. Most importantly, the ${\text{FAL}}_{2.5\text{mm}}$ protocol in combination with the FVCP protocol reduces the anteroposterior PTV margin to 7 mm and the mediolateral PTV margin from to 5 mm. Those in‐plane PTV margins are comparable to the 5 mm PTV margins applied in simultaneous integrated boost breast radiotherapy.^(6)^ Improved dose conformity in WBRT gains importance due to the renewed interest in hypofractionated treatment schedules. Evidence‐based clinical data on the heart toxicity in hypofractionated WBRT are, however, lacking. It is therefore recommended by the American Society for Radiation Oncology to restrict hypofractionation to patients not receiving systemic treatment and to exclude the heart from the primary irradiation fields, provided that the breast coverage is not compromised.^(18)^ Our proposed margin reduction might be the first step towards clinical trials to gain evidence on the applicability of hypofractionation schedules for a larger group of breast cancer patients.

Our proposed PTV margins are acceptable in WBRT. In contrast, in case of additional regional LN irradiation — improving disease free and metastases free survival in stage I‐III breast cancer patients^(1)^ — tight PTV margins are vital to maximally spare the heart and/or the ipsilateral lung. Authors de Boer et al.^(19)^ reported further reduction of the anteroposterior, mediolateral, and superior–inferior PTV margins to 2 mm, 2 mm, and 3 mm, respectively, by application of the extended no‐action–level (eNAL) protocol.^(20)^ This protocol eliminates time trends by recalculation of the systematic setup error based on weekly imaging and correction during the subsequent fractions. We did not observe growing residual setup errors during the course of treatment when the $\text{FVCP}-{\text{FAL}}_{2.5\text{mm}}$ protocol was applied. The measured residual setup errors in WBRT, starting at fraction 11, confirmed the expected (i.e., simulated) residual setup errors of the $\text{FVCP}-{\text{FAL}}_{2.5\text{mm}}$ protocol. However, further PTV margin reduction is possible by daily online setup correction based on tangential angled kV‐kV imaging. An isotropic 3 mm PTV margin accounts for the residual setup errors of the online setup correction protocol.^(12)^ Clinical implementation of daily setup correction is straightforward, as current linear accelerators are equipped with a kV source. Moreover, the imaging dose to the healthy tissue is negligible.^(12)^ However, we advise to combine daily online setup correction with the off‐line setup correction protocol proposed in this study. Applying off‐line setup correction after 5 treatment fractions significantly reduces the residual setup errors. The actual patient position being close to the intended patient position will facilitate and accelerate online matching during the subsequent fractions. In an upcoming study, the radiobiological impact of the off‐line and the online setup correction on target coverage and organs‐at‐risk sparing, respectively, will be quantified.

## V. CONCLUSIONS

The reproducibility of the anteroposterior patient setup in whole‐breast radiotherapy is influenced by the mobility of the skin marks relative to the bony matching anatomy. Fixed vertical couch position based patient setup eliminates the impact of the skin mark variability. The implementation of a small threshold for the correction of the systematic mediolateral and superior–inferior setup errors significantly reduces the number of off‐line setup corrections without deteriorating the setup accuracy.

## ACKNOWLEDGMENTS

The authors would like to express their appreciation to Prof. Dr. Peeters, Prof. Dr. Janssen, and Prof. Dr. Van Limbergen for their comments and suggestions. We would also like to thank the Myny‐Vanderpoorten Foundation for financial support.
